# Structural Heterogeneity Modulates Effective Refractory Period: A Mechanism of Focal Arrhythmia Initiation

**DOI:** 10.1371/journal.pone.0109754

**Published:** 2014-10-07

**Authors:** Martin J. Bishop, Adam Connolly, Gernot Plank

**Affiliations:** 1 Department of Biomedical Engineering, Division of Imaging Sciences & Biomedical Engineering, King's College London, London, United Kingdom; 2 Institute of Biophysics, Medical University of Graz, Graz, Austria; 3 Oxford eResearch Centre, University of Oxford, Oxford, United Kingdom; Gent University, Belgium

## Abstract

Reductions in electrotonic loading around regions of structural and electrophysiological heterogeneity may facilitate capture of focal triggered activity, initiating reentrant arrhythmias. How electrotonic loading, refractoriness and capture of focal ectopics depend upon the intricate nature of physiological structural anatomy, as well as pathological tissue remodelling, however, is not well understood. In this study, we performed computational bidomain simulations with anatomically-detailed models representing the rabbit left ventricle. We used these models to quantify the relationship between local structural anatomy and spatial heterogeneity in action potential (AP) characteristics, electrotonic currents and effective refractory periods (ERPs) under pacing and restitution protocols. Regions surrounding vessel cavities, in addition to tissue surfaces, had significantly lower peak downstream electrotonic currents than well coupled myocardium (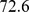 vs 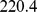 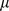
A/cm^2^), with faster maximum AP upstroke velocities (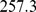 vs 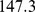 mV/ms), although noticeably very similar APDs (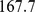 vs 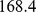 ms) and AP restitution properties. Despite similarities in APDs, ERPs in regions of low electrotonic load in the vicinity of surfaces, intramural vessel cavities and endocardial structures were up to 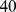 ms shorter compared to neighbouring well-coupled tissue, leading to regions of sharp ERP gradients. Consequently, focal extra-stimuli timed within this window of ERP heterogeneity between neighbouring regions readily induced uni-directional block, inducing reentry. Most effective induction sites were within channels of low ERPs between large vessels and epicardium. Significant differences in ERP driven by reductions in electrotonic loading due to fine-scale physiological structural heterogeneity provides an important mechanism of capture of focal activity and reentry induction. Application to pathological ventricles, particularly myocardial infarction, will have important implications in anti-arrhythmia therapy.

## Introduction

Recent advances in clinical electrophysiological mapping procedures, experimental optical mapping as well as computational modelling, have provided the ability to record and understand the nature of the electrical reentrant circuits which drive many types of cardiac arrhythmias. Such knowledge has highlighted the importance of structural anatomy in providing an essential substrate which stabilises reentrant circuits, particularly relevant in incessant ventricular tachycardia [1] and chronic atrial fibrillation [2]. However, despite our understanding of the stabilising effects of anatomical substrates during subsequent cycles of reentry, there remains a significant lack of knowledge regarding how the first reentrant cycle itself is initiated and the potentially important role structural anatomy, in both physiological and pathological scenarios, plays in this process.

Conduction block within slow conducting pathways or isthmuses of surviving tissue through ventricular scar has been shown to trigger reentry [3], which is thought to be a common phenomena in scar-related ventricular tachycardia. However, it is also thought in many cases that spontaneous activity from focal sources of tissue may provide the initial ectopic activation setting-up the initial reentrant wave. A problem exists, however, in that very large numbers of cells [4], [5] would need to be spontaneously activated in synchrony in order to successfully elicit a propagating wavefront - the triggered region needs to provide sufficient stimulating current to over-come the intrinsic electrotonic loading of the neighbouring tissue electrically-coupled to it, which acts to draw away depolarising current [6]. Such source-sink electrotonic mismatches may be mitigated by reductions in local electrical coupling [4], [7], [8], increases in anisotropy [7] or a decrease in tissue dimensionality at the point of the trigger [4] (for example, occurring in a pseudo-1D structures such as a Purkinje fibre), making successful capture more likely.

Electrotonic loading effects are known to influence many different aspects of cardiac electrophysiology [6]. During propagation, differences in wavefront curvature affect the amount of down-stream tissue any particular point on the wavefront must excite to propagate the activation [9]. Highly convex wavefronts - from focal sources, for example - and propagation across abrupt tissue expansions [9]–[11] often result in propagation failure and conduction block due to mismatches in electrotonic current source and sink. The cardiac action potential duration (APD) also decreases with distance from the pacing site due to electronic coupling between cells [12]–[15]. APD at the pacing site itself is correspondingly high as, during the recovery phase, all surrounding tissue is more depolarised than the initially activated region, causing an electrotonic flow of diffusive current that acts to prolong APD. Conversely, the presence of tissue boundaries reduces the APD of proximal tissue for wavefronts colliding with the boundary, due to the absence of more depolarised down-stream neighbours [12], [13]. Such a reduction in local electrotonic loading during wavefront boundary collision has also been shown to cause an increase in the local AP upstroke velocity as there is less loading of down-stream neighbours which require exciting [16], [17]. A consequence of this reduced loading is that tissue close to the boundary is more excitable, due to a reduction in the peak sodium current required to elicit an AP.

Structural anatomical heterogeneity within the myocardium, due to the presence of intramural blood vessels, endocardial structures such as trabeculae and papillary muscles, and extracellular cleft spaces, introduce the presence of boundaries and complex changes in cardiac fibre architecture that significantly affect local electrotonic loading. How the consequential effects of alterations in electrotonic loading in the vicinity of such fine-scale structural heterogeneities combine together to affect local refractoriness, excitability and the ability to successfully capture a focal triggered stimulus, is currently not well-understood. Furthermore, should a focal ectopic beat actually elicit propagation, it is also not clear the conditions under which the induced wavefront will become reentrant, requiring some form of conduction block or wavebreak to also occur, which will also be strongly dependent upon local structural anatomy and propagation pathways.

In this study, we aim to develop an in-depth, quantitative understanding of the biophysical processes by which fine-scale structural heterogeneities affect local electrotonic loading and the resulting direct effects on functional electrophysiological tissue properties and consequent arrhythmogenic implications. By using a combination of idealised and anatomically-detailed computational ventricular wedge models, we directly quantify fundamental relationships between electrotonic loading, refractoriness and successful capture of focal ectopics, and the dependence upon immediate structural heterogeneity, focussing on large sub-epicardial blood vessels, and also trabeculations. Our findings elucidate the important role played by physiological anatomical heterogeneity in arrhythmia initiation by ectopic mechanisms, which may have important implications in the setting of more significant pathological structural heterogeneity.

## Methods

### Computational Models

#### Model Geometries

Two separate unstructured, tetrahedral finite element meshes were used, each representing left-ventricular (LV) wedge preparations, but differing in their level of detail and complexity. The first model constituted a simplified, idealised representation of an LV wedge of cuboid dimensions 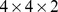 mm and containing a single, large sub-epicardial vessel of diameter 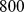 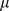
m, with centre a distance 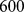 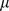
m from the epicardial surface. Mean element edge-length of the mesh was 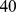 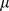
m. The second model constituted a highly anatomically-detailed LV wedge model derived directly from high-resolution rabbit MR data, as described previously [18]. Mean mesh discretisation of the anatomically-detailed model was 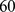 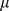
m. Both models contained a representation of a perfusing bath surrounding epi- and endocardial surfaces, filling all intramural cavities, with the bath also surrounding top and bottom surfaces. The idealised model therefore provides a less computationally-demanding model to conduct extensive simulations and analysis, as well as carefully control parameters relating to wavefront directions, whereas the anatomically-detailed model allows for an important validation of uncovered relationships.

#### Functional Model Parameterisation

Transversely-rotational fibre orientation was assigned to the models, rotating ±60° between epi- and endocardial surfaces. A previously described algorithm based-on a Laplace-Dirichlet approach [19] was used for assigning the smooth negotiation of cardiac fibres around intramural cavities, informed from histology [20]. The electrically-insulating effects of the connective tissue surrounding blood vessel walls was represented by assigning tagged elements around vessel cavities in the meshes with reduced electrical conductivity values derived directly from experiment [19].

Cell membrane dynamics within the myocardial tissue were represented by a recent rabbit ventricular cell model [21]. Conductivities along the fibre (*l*) and cross-fibre (*t*) directions within the intracellular (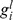, 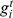) and extracellular (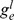, 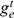) domains were defined by previous experimentally-obtained values [22]. Bath conductivity was set to 1.0 S/m, with vessel lumen wall conductivity 0.01 S/m.

### Simulating Cardiac Electrophysiology Dynamics

#### Governing Equations

Electrical activation throughout the models was simulated based on the bidomain equations [23]

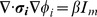
(1)

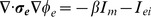
(2)

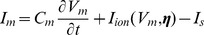
(3)

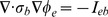
(4)where 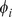 and 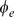 are the intracellular and extracellular potentials, respectively, 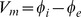 is the transmembrane voltage, 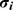 and 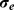 are the intracellular and extracellular conductivity tensors, respectively, 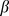 is the membrane surface to volume ratio, 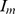 is the transmembrane current density, 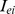 and 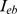 are extracellular stimuli applied in the interstitial space or the bath, respectively, 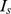 is a transmembrane stimulus, 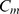 is the membrane capacitance per unit area, and 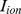 is the membrane ionic current density which depends on 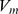 and a set of state variables 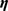. At tissue boundaries, no flux boundary conditions are imposed for 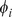, with 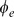 being continuous at the interface. At the boundaries of the conductive bath surrounding the tissue, no flux boundary conditions for 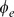 are imposed.

In certain scenarios, the monodomain representation was used whereby the cardiac tissue is represented as a single conducting domain and the bidomain equations reduce to the monodomain equation, with conductivity given by the the harmonic mean conductivity tensor or the effective bulk conductivity (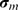) [24]. Monodomain simulations were used in the case of the high-resolution wedge model when performing simulations to measure focal electrotonic loading and refractory period (described below) due to the significant computational burden these simulations represent upon a mesh of such detail. In the case where monodomain was used, the augmented formation was not used as when using the Clerc conductivities (as used here) the effects of bath-loading on the wavefront are minimal.

#### Computational Considerations

The bidomain equations were solved with the Cardiac Arrhythmia Research Package (CARP) [25]. The specifics of the numerical regimes used in CARP have been described extensively elsewhere [25]–[27]. Visualisation of results was performed with the custom written Meshalyzer software (courtesy of Dr Edward Vigmond).

### Stimulation Protocols & Analysis

All protocols described below were applied to the idealised wedge model, with selected protocols also applied to the anatomically-detailed model.

#### Measuring Electrotonic Loading During Propagation

Two separate wavefront propagation directions were considered: circumferential (initiated by stimulus of a transmural tissue plane) and transmural (initiated by stimulus of the entire endocardial surface). A basic cycle length (BCL) of 400 ms was used for 10 paced beats to achieve steady-state. Electrophysiological metrics were then quantified on the 11th beat. APDs were defined as the time-point between depolarisation (positive crossing of 0 mV threshold) and 90% repolarisation and were calculated at all node points within the models. Maximum AP upstroke velocity was also calculated for all nodes as the maximum positive rate of rise during depolarisation with an output time discretisation of 0.1 ms.

**Figure 1:**
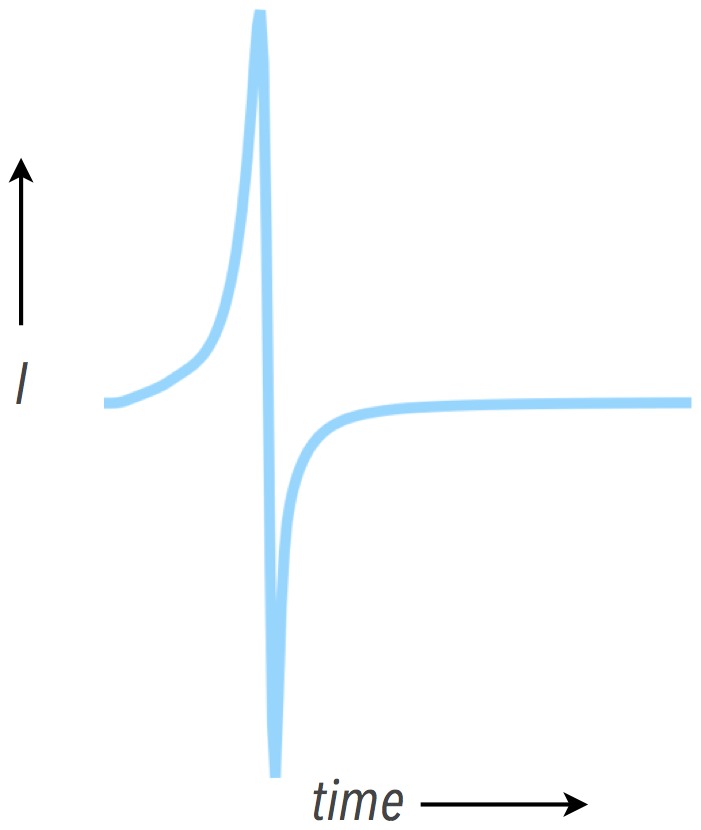
Representative total electrotonic current from a point in the tissue during normal propagation demonstrating the biphasic profile.

Total electrotonic current at each node was calculated directly from the diffusion term of the bidomain equation 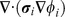. In the finite element regime used here, this involved scaling the product of 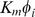 (where 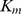 is the stiffness matrix) with 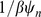 where 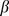 is the surface-to-volume ratio and 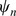 is the hat function associated with the node point. The units of total electrotonic current were therefore *µ*A/cm^2^. The calculated electrotonic current is equivalent to the total transmembrane current, 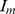, representing the difference between the total inward and total outward current seen by the node i.e. the difference between current entering and leaving the cell via gap junctions. During propagation, the total electrotonic current has a biphasic profile, as shown in Figure 1, with the initial positive inflection representing greater current flux into the cell than leaving the cell and the second negative inflection representing greater current efflux away from the cell than current entering the cell. In terms of membrane-level events, the first phase of the biphasic electrotonic profile can also be thought of as the capacitive charging of the membrane, with the subsequent negative phase largely due to the engagement of the fast sodium current. The peak maximum positive electrotonic current therefore provides a representation of the degree of upstream electrotonic loading (current received from a local region from its upstream neighbours as it acts like a sink), with the peak negative electrotonic current representing the degree of downstream electrotonic loading (current passed-on by the local region to its downstream neighbours as it acts like a source).

Finally, the effects of restitution on the above electrophysiological metrics was performed by progressively decreasing BCL 400 ms to 170 ms, until loss of capture, with 10 paced beats performed at each BCL. Progressively decreasing increments were used: 50 ms (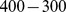 ms), 20 ms (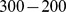 ms), 10 ms (200− loss of capture).

#### Measuring Focal Electrotonic Loading

As described above in Section, information regarding electrotonic loading may be obtained from considering peak positive and peak negative electrotonic current flow associated with a cell or region of tissue. However, these quantities are defined and calculated during propagation, and are therefore very sensitive to respective wavefront propagation directions. In order to derive a single quantitative measure of focal electrotonic loading (current-sink), we realise that sites with a high electrotonic loading ‘share’ a large proportion of the current used to stimulate them with surrounding tissue. Thus, during focal stimulus, maximum changes in 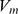 from rest at the stimulus site itself can be related directly to the local degree of current-sink associated with that site. Successive point transmembrane stimuli were applied to individual tetrahedral finite elements within the models and the mean change in potential 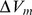 from rest within the element recorded. Tissue electrophysiology was represented by a passive membrane model to avoid active recruitment of ion channel dynamics and initiating active propagation. Resting membrane potential was set to 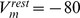 mV (approximate resting potential of rabbit ventricular myocardial cells) and membrane resistance 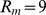 k
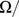
cm^2^ [8] in the passive model. Continuous stimuli were applied for 20 ms, as in [8], loading the membrane to 90% of its final value in steady-state i.e. until 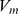 reached 90% of its steady-state value. All four nodes of each tetrahedral element were stimulated to mitigate potential differences in total applied stimulus current due to variations in the volume of the nodal hat functions with spatial variation of element size. This therefore necessitated computed values to be plotted on element centroids. To ensure further accuracy, the total injected current source density was scaled such that the same total current was injected into the tissue in each simulation. Stimulations were performed sequentially, such that one simulation was performed for each element stimulated. Steady-state values of 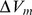 associated with each stimulated element (mean 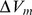 value of all 4 nodes) were then calculated.

#### ERP Protocol

Effective Refractory Period (ERP) is defined as the minimum time (coupling interval, CI) after being fully activated (eliciting an AP) that a region of tissue may once again capture a focal stimulus and initiate another propagating wave of activity. Successive stimuli were applied to small regions of tissue within the models (approximately 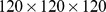 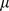
m) to measure ERP following a transmural preconditioning S1 beat. The strength of the stimulus used in the ERP protocol was defined to be the minimum stimulus strength required to elicit propagation within a region of well-coupled tissue at rest (default strength 700 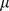
A/cm^2^, duration 2 ms); a strength of less than this would not compute an ERP as propagation would never be inducible within parts of the domain. The initial state of the tissue for ERP calculation in both models was as following a transmural propagation stimulus at a BCL of 170 ms. In all cases, ERP was defined relative to the prior activation time at each individual focal region following activation by the preceding S1 transmural wavefront propagation.

**Figure 2:**
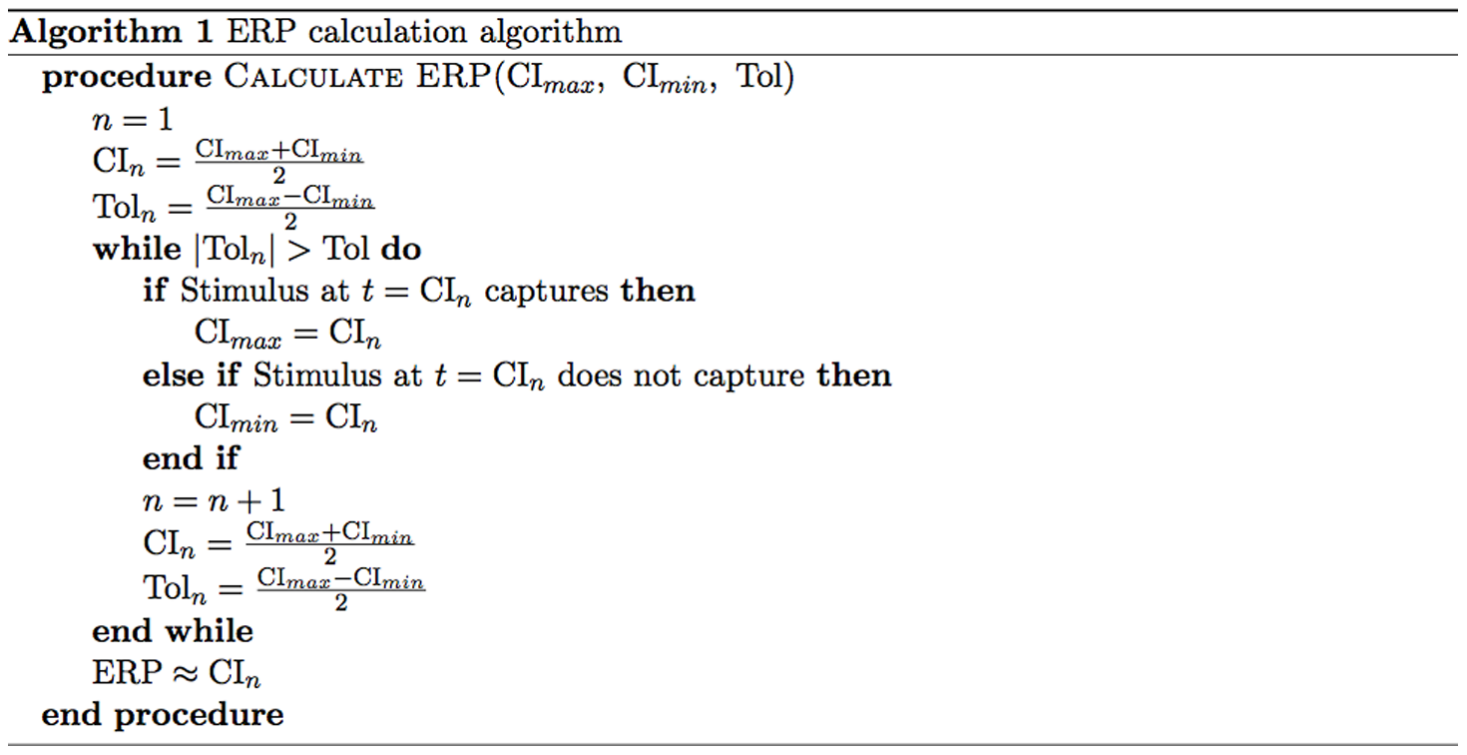
Algorithm used to calculate ERP.

Instead of using progressively shorter CI, a more computationally efficient *divide and conquer algorithm* was used. Initially, an upper limit (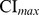) was defined as a large CI after which the tissue region will be completely recovered and will certainly capture another focal stimulus (100 ms following recovery to 90% of its initial resting potential value, for example). A lower limit (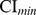) was then defined as a small CI at which the tissue will definitely not capture when stimulated (20 ms prior to the tissue returning to 90% of its initial resting potential value, for example). The ERP therefore lies within 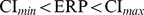. The divide and conquer algorithm works by initially stimulating at a trial CI at the mid-point of the max and min limits 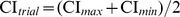. If capture occurs, then the maximum CI value is updated to this trial value, 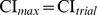; should capture not occur, the minimum CI value is updated to the trial value, 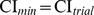. The next trial then occurs at the new mid-point between the new 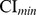 and 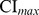. The true ERP can always be estimated as occurring at the mid-point between 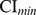 and 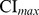. After *n* successive trials, the ERP can thus be estimated within an accuracy of 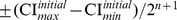. Here, an initial window of 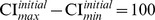 ms was used with with 5 iterations, ensuring accuracy in the estimated ERP of 1.6 ms. The algorithm used to calculate ERP is summarised in Figure 2.

#### Reentry Induction

In scenarios in which specifically chosen S2 stimuli resulted in the generation of reentry, identification of filaments (the organising centres of reentrant activity) was performed using a previously described approach [28].

## Results

### Electrotonic Loading During Steady-State Pacing

**Figure 3:**
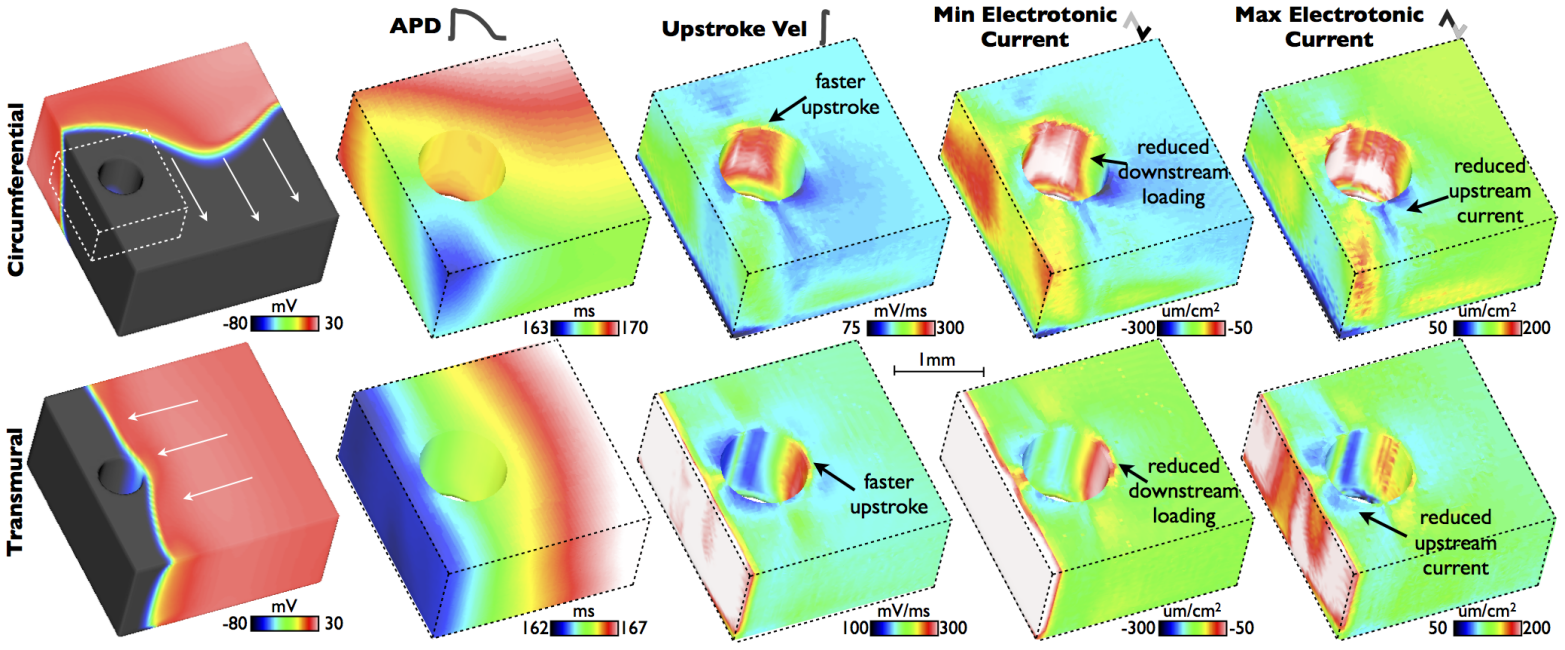
Steady-state spatial properties of APDs, max upstroke velocities and peak electrotonic currents around the simplified vessel model for transmural (top panels) and circumferential (bottom panels) propagation directions. Left-hand panels show snap-shots of *V_m_* distributions during respective propagation. White dashed box shows the region of interest around vessel cavity highlighted in data panels, with upper-surface being along the mid-plane through the tissue. A scale-bar is shown for the right-hand four data columns.

Pacing protocols were applied to both the idealised and anatomically-detailed models, as described in Section. Figure 3 shows spatial plots of APD, max upstroke velocity and peak negative and positive electrotonic currents within a highlighted region of the idealised wedge model during pacing at a BCL of 400 ms (data shown for the 11th paced beat). APD is seen to decrease steadily and by only 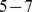
ms along the direction of pacing, although with little noticeable change or heterogeneity in the vicinity of the vessel cavity. In contrast, max upstroke velocity and peak electrotonic currents are relatively homogeneous throughout the domain, but show a distinct region of strong heterogeneity both around the vessel cavity itself and at other boundaries upon which the wavefront collides. Specifically, max upstroke velocity shows a strong increase at the proximal side of the vessel cavity where the wavefront collides with the cavity boundary, increasing by approximately 3-fold relative to well-coupled tissue away from boundaries. A similar increase in max upstroke velocity is also seen at the epicardial surface during transmural pacing. Max upstroke velocity decreases at the distal side of the vessel cavity, to a lower value than in well-coupled tissue distant from the cavity. Peak negative electrotonic current shows a very similar spatial distribution and magnitude of change to max upstroke velocity, although in this case peak negative current is lower at sites corresponding to wavefront collision with boundaries. Peak positive electrotonic current shows an approximately similar spatial distribution to max upstroke velocity, being higher at sites corresponding to wavefront collision with boundaries, although with smaller magnitude of change. It is important to note that the spatial changes highlighted in Figure 3 are all relatively confined to within 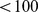 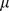
m of the boundaries.

**Figure 4:**
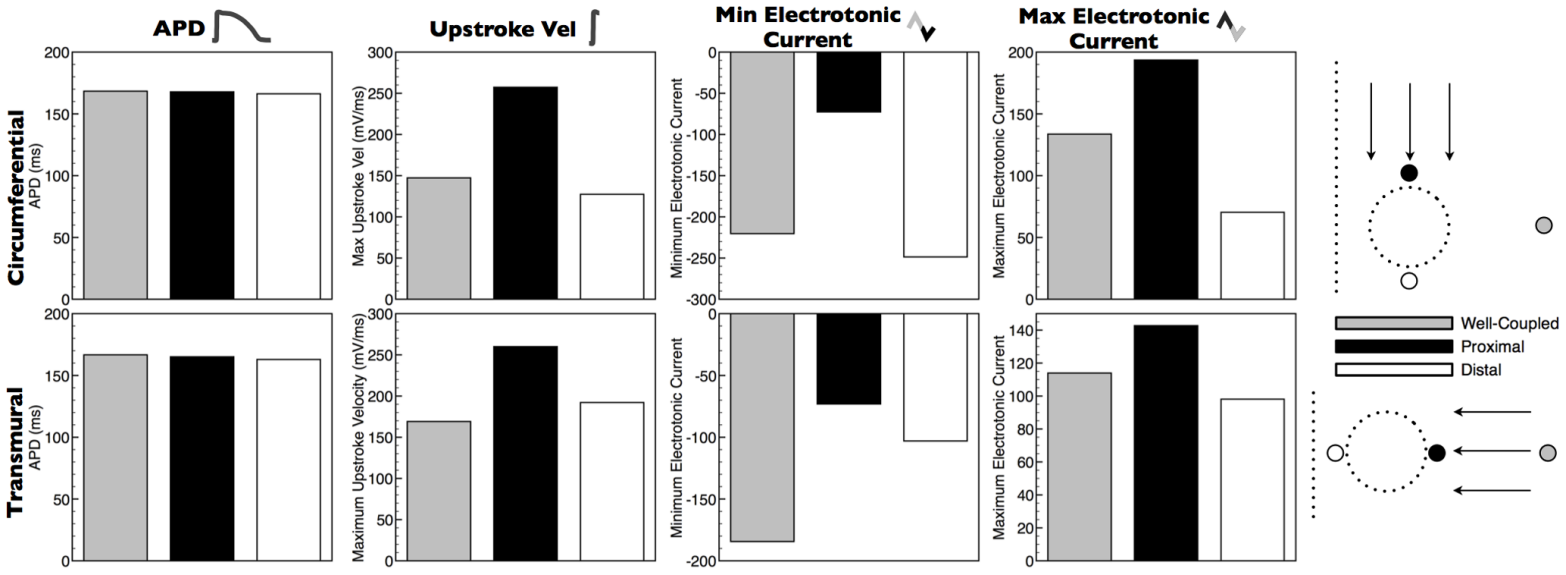
Steady-state values of APDs, max upstroke velocities and peak electrotonic currents at 3 points representing regions experiencing different electrotonic loading with respect to wavefront propagation directions. Proximal and distal nodal locations represent points immediately on the cavity boundary at points spanning the diameter of the cavity. Respective labelling is as shown in the schematic for each propagation direction. In both cases, the well-coupled point is located 2000* µ*m from the epicardial surface at the centre point of the tissue.

Figure 4 quantifies the results shown in Figure 3, explicitly comparing numerical values of APD, max upstroke velocity and peak positive/negative electrotonic current for nodal points proximal and distal to the vessel cavity with respect to wavefront collision, as well as a well-coupled point far away from any boundaries. For both propagation directions, APDs are very similar between all points being 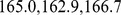 ms, respectively, for proximal, distal and well-coupled points for transmural propagation and 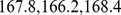 ms for circumferential propagation. Max upstroke velocity, however, is approximately 35% faster at the proximal node (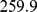 mV/ms), compared to the distal node (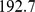 mV/ms), and approximately 53.2% faster than the well-coupled node (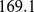 mV/ms) during transmural pacing. During circumferential pacing, however, larger changes are seen with max upstroke velocity being approximately twice as fast at the proximal node (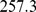 mV/ms), compared to the distal node (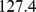 mV/ms), and approximately 75% faster than the well-coupled node (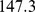 mV/ms). Peak negative electrotonic current is significantly reduced at the proximal node relative to the well-coupled node in both pacing directions, being 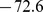 vs 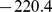 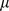
A/cm^2^ (67% reduction) for circumferential propagation and 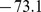 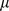
A/cm^2^ vs 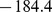 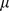
A/cm^2^ (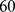
% reduction) for transmural propagation. Interestingly, the distal node is seen to have a higher peak negative electrotonic current (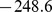 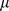
A/cm^2^) than the well-coupled node during circumferential propagation, but a lower value (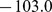 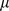
A/cm^2^) during transmural propagation. The peak positive electrotnic current is almost 3-fold larger at the proximal node relative to the distal node for circumferential propagation (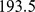 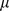
A/cm^2^ vs 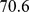 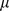
A/cm^2^), whereas this difference is only 45% for transmural propagation (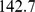 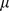
A/cm^2^ vs 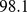 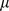
A/cm^2^). Comparing with the well-coupled node, the peak positive electrotonic current is 45% larger (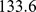 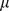
A/cm^2^) and 26% (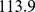 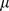
A/cm^2^) for circumferential and transmural propagation directions, respectively.

**Figure 5:**
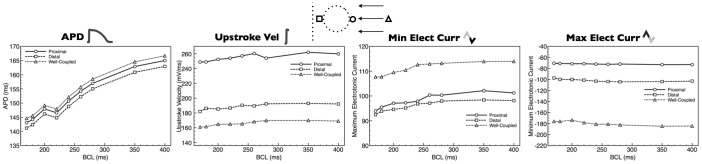
Steady-state spatial properties of APDs, max upstroke velocities and peak electrotonic currents around the anatomically-detailed wedge model, for transmural (top panels) and circumferential (bottom panels) propagation directions. Inset regions highlight changes in the vicinity of sub-epicardial vessels, with corresponding scale-bar. Left-hand panels show snap-shots of *V_m_* distributions during respective propagation.

A similar pacing protocol was conducted on the anatomically-detailed wedge model. Figure 5 shows spatial distributions of APDs, upstroke velocities and peak positive/negative electrotonic currents within the MR-based model at steady-state for a BCL of 300 ms. Two separate regions in the vicinity of large sub-epicardial blood vessels are highlighted in each case. Qualitatively similar results can be seen to the case of the idealised model, with proximal regions of tissue corresponding to wavefront collision with vessel cavities showing increased max upstroke velocities and reduced peak negative currents, with regions distal to wavefront collisions showing reduced peak positive electrotonic current. In all cases, these changes are confined to tissue within approximately 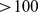 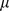
m of the boundaries. Again, similar to the idealised model, although dispersion of APD was seen along the pacing direction, no significant APD variation was witnessed in the vicinity of structural heterogeneities.

**Figure 6:**
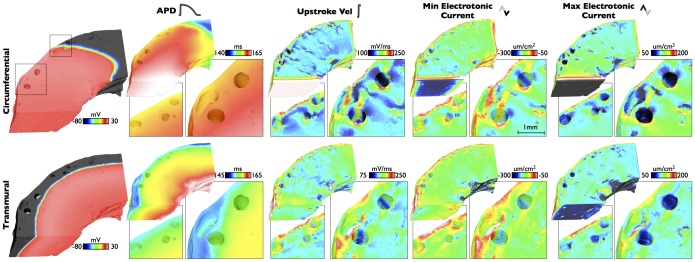
Restitution properties of APD, max upstroke velocity and peak electrotonic currents as BCL is decreased from 400 ms to 170 ms at 3 points representing regions experiencing different electrotonic loading during transmural wavefront propagation.

Restitution of the changes in electrophysiological properties noted above were then examined in the simple model. Figure 6 shows changes in APD, max upstroke velocity and peak electrotonic currents at a proximal and distal node with respect to wavefront propagation directions, in addition to at a well-coupled node away from tissue boundaries (node locations defined as in Figure 4), as BCL is progressively decreased in the case of transmural propagation. APD is seen to decrease progressively with BCL, as expected, with the appearance of alternans in APD noticeable below 230 ms BCL. However, interesting, no relative difference is seen in restitution effects between points with different electrotonic loading properties. Max upstroke velocity and peak negative electrotonic current both show slight restitution effects, decreasing by less than 10% over the entire protocol. Similarly to the case of APDs, no relative differences in restitution effects are witnessed in either max upstroke duration or peak negative electrotonic currents. Peak positive electrotonic current shows very little noticeable restitution effects across all nodes. Similar findings were seen for circumferential wavefront propagation directions.

### Relation to Focal Electrotonic Loading

**Figure 7:**
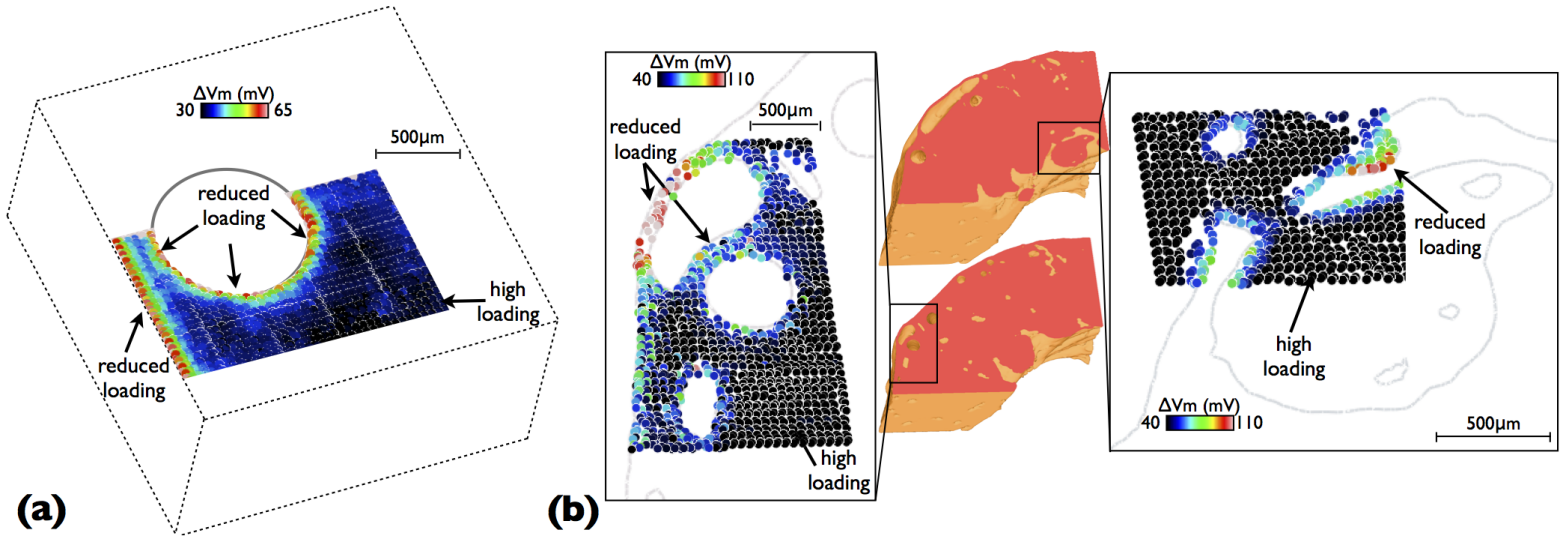
Maximum change in *V_m_* from rest (Δ*V_m_*) following stimulation of individual tetrahedral elements in a passive tissue model. Data is displayed on element centroids and is only shown around the region of interest for the idealised model (a) and the high-resolution wedge model showing (left) around large subepicardial blood vessels and (right) around endocardial trabecular structures.

The stimulation protocol described in Section was applied to both idealised and anatomically-detailed wedge models to quantify focal electrotonic loading, focussing on small regions in the neighbourhood of high structural heterogeneity in each case. Figure 7(a) shows the resulting plot of maximum change in 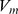 in the idealised model, with data displayed on element centres (centroids). Focal electrotonic loading is seen to be significantly reduced in the vicinity of vessel cavities and tissue surfaces, with over a 2-fold increase in 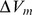 seen relative to well-coupled tissue. These changes in focal electrotonic loading seem to be relatively confined to 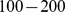 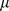
m of the boundaries. However, due to the proximity of the sub-epicardial vessel represented in the idealised model to the epicardial surface, the narrow channel of tissue between the vessel cavity and the epicardium experiences a strong reduction in electrotonic loading, affecting a relatively large region.

Figure 7(b, left) shows the spatial variation in focal electrotonic loading, characterised by changes in 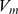 following individual focal, sub-threshold stimuli in the vicinity of a number of large sub-epicardial vessels in the anatomically-detailed wedge model. Again, similar to the idealised model, 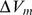 is largest close to the boundaries of vessels as well as the epicardial surface. In all cases, these changes are confined to tissue within approximately 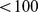 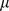
m of the boundaries. In the region examined, however, the larger vessel is particularly close to the epicardial surface, leaving a particularly narrow region of myocardium between the vessel cavity and the surface. In this region, 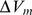 is seen to be larger than in other regions of tissue close to boundaries. Such an effect is more prominent here than in the case of the simple model above. Similar increases in 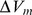 are also seen in thin regions of tissue separating blood vessels.

Finally, Figure 7(b, right) also shows a similar plot of 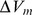, but this time in the vicinity of a trabecula structure, attached to the endocardium by a narrow piece of tissue. Interestingly, we note that, although 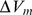 is slightly raised along the surfaces, as seen before, no particular increase is seen in the narrow region of attachment. However, a reduced electrotonic loading effect is seen to be more evident in the highlighted region corresponding to a small (well-attached) trabecula ridge, with a narrow radius.

### Effective Refractory Period

**Figure 8:**
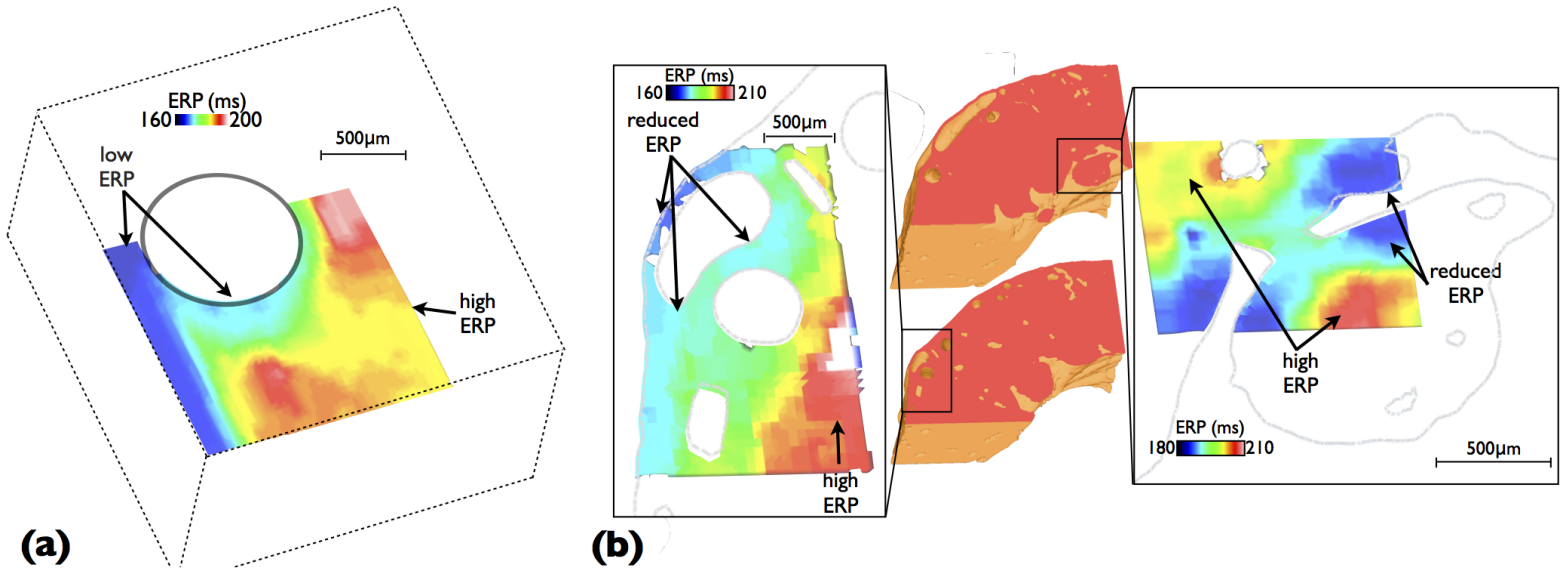
Spatial variation in ERP in (a) the immediate vicinity of vessel and tissue boundary within the idealised model, and (b) regions around large subepicardial blood vessels (left) and around a large endocardial trabecula structure (right). Here, the S1 preconditioning stimulus was in the transmural propagation direction.

ERP was computed using the algorithm described in Section at a number of different spatial locations surrounding the blood vessel cavity in the idealised model, the results of which are plotted in Figure 8(a). Immediately obvious in Figure 8(a) is the significant reduction in ERP both in the vicinity of the vessel cavity and close to the epicardial surface. In these regions, ERP is some 40 ms shorter than in regions of well-coupled myocardium away from all boundaries. Such reductions in ERP are seen to occur over relatively larger spatial regions of tissue than those changes seen in upstroke and electrotonic currents during propagation. ERP is lowest (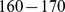 ms) in a ‘channel’ of tissue between the epicardial surface and the vessel cavity, although it is also relatively low (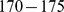 ms) on the bottom side of the vessel cavity, extending some 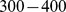 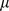
m into the tissue. Interesting, ERP is seen to decrease more rapidly in a transmural direction away from the cavity (approximately perpendicular to the local fibre orientation) compared to in a circumferential direction (approximately parallel to the local fibre orientation). Regions of stark transition between high and low ERP are also noted to occur between the channel of low ERP, moving into the well-coupled myocardium.

Figure 8B shows an similar plot of the ERP, but this time computed within two separate confined regions close to the epicardium (left) and the endocardium (right) within the anatomically-detailed wedge model similar to those shown in Figure 7(b). The epicardial region contains a number of large subepicardial blood vessels. Here, again ERP is characteristically lower both close to the epicardial surface and within the narrow channel of tissue between the large subepicardial vessels and the epicardium, as seen above, although in this case ERP is reduced by even more due to the narrow nature of the channel compared to Figure 8(a). In addition, we note that the complex anatomy and vasculature network highlights that an additional area in which ERP is substantially lowered lies within the narrow channel of myocardium separating two vessel cavities.

The spatial distribution of ERPs close to the endocardium shows a slightly lower range of values compared to that seen at the epicardium, as there are no large vessels to create narrow channels of tissue. As expected, ERP is low close to the surface boundaries, both the endocardium itself and also the surface of the trabecula However, ERP is seen to increase as these surfaces join together at the narrow strand of tissue which joins the trabecula to the endocardium. Not only is ERP then significantly higher within the mid-myocardium, but it is also seen to increase to similar levels within the bulk of the trabecula itself, which represents a relatively thick structure approximately>1 mm in diameter. Consequently, this results in significant heterogeneity in ERP within this relatively small spatial region.

### Arrhythmogenic Effects of Reduced Electrotonic Loading

**Figure 9:**
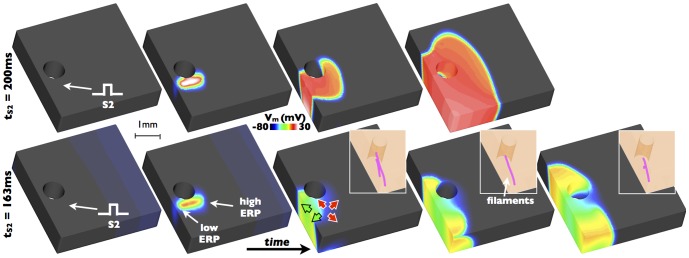
Induction of reentry and the formation of filaments (shown as pink lines) following a premature S2 stimulus applied 163 ms following the prior activation (bottom) along with uniform capture following an S2 stimulus applied after 200 ms (top).

Figure 9 demonstrates how the highlighted heterogeneity in ERP, mediated-by electrotonic loading variations due to the presence of the blood vessel cavity, may induce non-uniform conduction block and reentry. Both panels of Figure 9 show the application of a focal stimulus to the same region of tissue identified in Figure 8 to lie within a high gradient of ERP, proximal to the vessel. The stimulus in the upper panel is applied some 200 ms after prior activation of the tissue region following transmural propagation, and thus the tissue region stimulated, as well as all other tissue surrounding it, is fully recovered. Consequently, uniform capture of this focal beat occurs and no reentry is formed. However, the lower panel of Figure 9 shows the case where the S2 stimulus is applied prematurely, just some 163 ms following the initial activation. Because the premature pulse is applied across a region of high ERP gradient, the tissue surrounding the stimulus site proximal to the vessel cavity and to the epicardial surface is able to be successfully excited by the stimulus, due to the relatively lower ERP in this ‘channel’ with respect to the stimulus timing, as seen in Figure 8. In contrast, the tissue towards the intramural side of the stimulus site represents well-coupled myocardium which has a relatively higher ERP (from Figure 8) relative to the timing of the pulse, meaning capture of tissue in this direction does not occur. The overall effect is to induce non-uniform conduction block, with the wavefront propagating towards the epicardium and the vessel cavity (shown by green arrows in Figure 9) whilst blocking in the direction into the tissue towards the endocardium (shown by red arrows in Figure 9). An additional consequence of this is the formation of filaments (3D phase singularities) at the site of initial block, whereby the wavefront initially pivots around a region of sharp ERP gradient. It is noted, however, that this case of reentry was not sustained and was extinguished very shortly after its initiation.

Simulations were also conducted in which tissue was stimulated at the same timing as the above case in which reentry was initiated (163 ms), but in locations where ERP was relatively uniformly high (in the region of mid-myocardium, towards the endocardial side of the tissue) and low (on the epicardium, near the vessel). The former case resulted in the stimulus failing to capture at all, as all tissue near the stimulus site was not yet recovered, whilst the latter case lead to the stimulus capturing relatively uniformly and eliciting wavefront propagation that did not initiate reentry.

## Discussion

Although much knowledge exists regarding how anatomical substrates stabilise and support reentrant activity, little is currently known about the role structural anatomy plays in the genesis of the initial trigger that establishes the first reentrant beat. Particularly, it is not well understood how the small isolated regions of tissue which provide the trigger may successfully over-come intrinsic current source-sink mismatches to elicit wavefront propagation into the surrounding tissue, and subsequently setup a reentrant circuit. In this study, we have used anatomically-detailed computational models to demonstrate how physiological structural heterogeneity significantly reduces local tissue electrotonic loading, directly affecting tissue excitability, providing an important substrate to facilitate the capture of focal activity and initiation of reentry.

### Effect of Electrotonic Loading on Electrophysiological Metrics During Propagation

The detailed modelling performed in this study has allowed us to quantify and understand the biophysical processes by which electrotonic loading is reduced by the presence of fine-scale structural features - such as intramural blood vessels and endocardial trabeculations - due to an increase in intracellular boundaries and a corresponding localized change in the ratio between boundary and myocardial volume. During wavefront propagation, the presence of structural heterogeneity was seen to have little affect on APD, with the expected dispersion of APD along the pacing direction dominating [12]–[15]. However, maximum upstroke velocity was seen to be significantly increased close to the tissue boundaries (including intramural cavities and epi/endocardial surfaces) on the proximal side of wavefront collision, as suggested previously for epicardial boundaries [16], [17]. This spatial pattern of reduced upstroke velocity was similar to that of the reduction in peak maximum negative electrotonic current (Figures 3 & 5) in the same area, suggesting it is directly driven by a reduction in downstream electrotonic loading due to the presence of the intracellular boundary (represented by vessel cavity or epi/endocardial surfaces). For tissue on the proximal side of the boundary, as less outward current is leaving due to reduced loading, the maximum positive electrotonic current correspondingly reaches a higher value on the proximal side of the cavity or boundary; also a direct consequence of the faster upstroke velocity in this region. Important to note is that these effects on upstroke velocity and electrotonic currents appear to be confined to within approximately 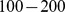 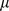
m of the boundary, and also show little effects of restitution with existing differences between regions being maintained with changes in BCL.

Differences were seen in the absolute magnitude (Figure 4) and the spatial patterns (Figure 3) of these metrics between the cases of transmural and circumferential pacing. We believe theses differences to arise from the interaction of the wavefront with the complex fibre architecture in these regions, which it will do so in a different manner for these two different pacing directions. Around vessel cavities, the fibre architecture is very complex, with the fibres smoothly negotiating around the vessel, as demonstrated in our earlier work [19] and represented in the models used in this study. The degree of electrotonic loading on the activation wavefront depends on its direction of propagation with respect to the primary fibre direction due the anisotropic conductivity. It also depends on the local wavefront curvature which may itself depend upon both the wavefront's prior propagation history and the local fibre orientation. Together these combined affects of anisotropy lead to noted differences in the effects of electrotonic loading on the measured metrics.

### Quantification of Local Electrotonic Loading During Focal Stimulus

Simulation of paced wavefronts allow active quantities such as upstroke durations and electrotonic currents to be explicitly quantified and their spatial distribution with respect to structural heterogeneity examined. In this context, Figures 3, 4 & 5 demonstrated that although such metrics are useful in identifying regions of low electrotonic loading, they were only able to do so in a manner that was highly dependant upon activation sequence, effectively identifying regions where the loading was reduced upon the propagating activation wavefront where it collided with boundaries and cavities. However, of key relevance for the capture of focal ectopic beats is the *focal* electrotonic loading, a quantity which depends upon tissue conductivities, fibre orientation and presence of boundaries within a localised three-dimensional volume surrounding the focal source. Due to the highly complex nature of the models used here, we sought to develop and apply an elegant measure of focal loading as the change in 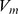 from rest at the stimulus site upon point stimulation with a passive electrophysiological model. Performing such simulations was highly computationally expensive, as individual simulations need to be performed to derive this focal loading metric at each point. Although consequently confined to small regions within the models, these simulations provided important information, not available during pacing. Firstly, the findings from these simulations again highlighted the reduction in electrotonic loading in the vicinity of structural boundaries, including vessel cavities and exterior surfaces. However, they also highlighted the significant reduction in electrotonic loading in other key areas, such as within the ‘channel’ of tissue between large sub-epicardial vessels and the epicardium itself, between two neighbouring vessels within close proximity, and also within small trabeculations and ridges (see Figure 7(a) & (b)). In the latter case, it should be noted that the effects of local tissue curvature play an important role with the reduced loading in the narrow trabecular ridge potentially being partially attributed to the narrowing of the tissue at this point. As was the case with the active metrics above, such reductions in focal loading were confined to regions close to the boundary.

### Electrotonic Loading-Mediated ERP Heterogeneity

Of key relevance to the interpretation of the above findings lies in linking this reduction in electrotonic loading, driven by structural heterogeneity, to its functional consequences in terms of capture of focal stimuli. A highly-useful clinical measure of the susceptibility of a region for capture of a focal stimulus is in the determination of the ERP for point stimulation. In this study, for the first time, we computed the spatial variation of ERPs around key areas of high structural heterogeneity within highly anatomically-detailed models, made possible through the development of a computationally-efficient ERP protocol for use at the tissue level. Despite seeing very little variation in APD and repolarisation times in the vicinity of fine-scale structural features during wavefront propagation, significant heterogeneity was witnessed in ERP. The large reduction in ERP around structural features and tissue boundaries is, we believe, primarily explained by the changes in the excitability of the tissue in these regions, introduced by the close proximity of intracellular discontinuities. The corresponding reduction in electrotonic loading in these areas means that, upon stimulation with a given strength of current, less of the stimulating current diffuses away to neighbouring tissue, meaning that more is able to be used by the stimulated region itself to help bring it to threshold and depolarise. The tissue in these regions is therefore more excitable, with a corresponding lower ERP.

**Figure 10:**
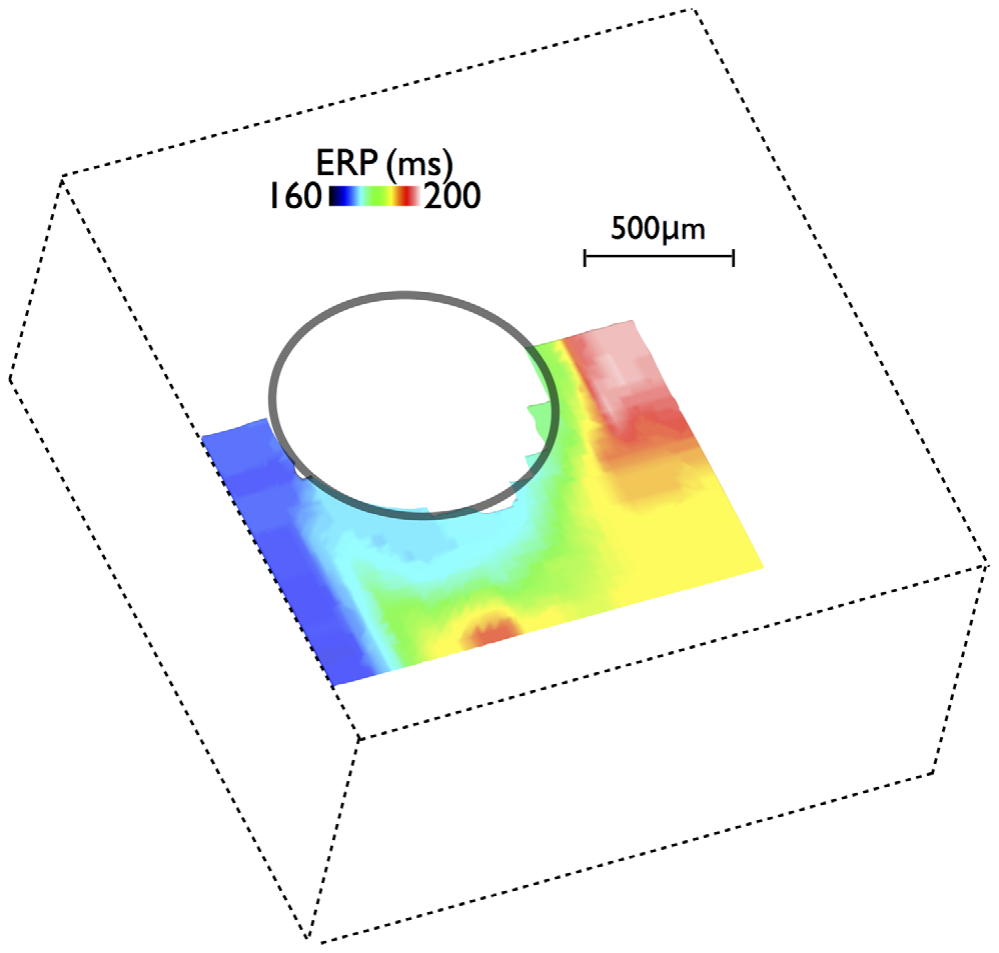
Spatial variation in ERP in the immediate vicinity of vessel and tissue boundary within the idealised model, following an S1 preconditioning stimulus applied in the circumferential direction.

The ERP protocol used in this study used a transmural stimulus as its S1 pre-conditioning pulse. As noted above, although there was no noticeable heterogeneity in APD surrounding the vessel cavity (following either the transmural or circumferential stimulus), there was a minor gradient in APD (changing by just a few ms) along the direction of wavefront propagation (Figure 3). In the case of transmural propagation, this resulted in regions of low APD occurring close to the epicardial surface, with regions of longer APD occurring within the mid-myocardium, thus bearing some similarity with the spatial pattern of ERP variation in Figure 8(a) (although not in the vicinity of the cavity). This consequently raises the questions as to whether these, albeit relatively minor differences in refractoriness, are driving the noticed differences in ERP, or whether it is due to the differences in electrotonic loading due to proximity of boundaries, as discussed above. To address these issues, we repeated the ERP protocol within the simplified model, but this time using the circumferential stimulus as the S1 preconditioning pulse, shown in Figure 10. The spatial pattern of ERP was almost identical to the case of the transmural S1 stimulus, underlining the fact that the minor differences in repolarisation in the direction of wavefront propagation following the S1 do not play an important role in governing the boundary-induced heterogeneity in ERP uncovered in this study.

Due to the high anatomical complexity of the network of coronary vessels and endocardial structures represented within the models, very high gradients in ERP occurred within close proximity to one-another. Specifically, differences of approximately 40 ms in ERP (approximately 25% of APD) were seen within spatial regions of approximately 1 mm, as witnessed in Figures 8(a) & (b). As such, the spatial distribution in ERPs was seen to affect regions further from the tissue and cavity boundaries than in the case of the other metrics discussed above. We believe this to be due to the fact that the electrotonic effects upon both the refractoriness of the tissue and its excitability are combined together in ERP, affecting regions further from the boundary. The main regions of stark ERP heterogeneity were similar to those identified previously as regions of low electrotonic loading: in the narrow region of tissue both between large sub-epicardial vessels and the epicardium as well as between two separate neighbouring vessels, in addition to around trabeculations close to their attachment to the endocardial wall.

It should be noted here that ERP also depends upon the specific nature of the protocol used in its calculation. For example, stimulating with a stronger stimulus or over a larger tissue volume would have caused a reduction in ERP values. Here, we carefully selected the combination of smallest feasible tissue volume and lowest (physiological) stimulus strength to just elicit propagation in the regions of well-coupled myocardium, thus most closely resembling a physiological scenario of stimulus due to a mechanism such as a spontaneous earlyafter depolarisation. Finally, it should also be noted that the previously mentioned effects of complex fibre orientation also appears to have an impact on ERP. This is thought to be responsible for the slight heterogeneous variation in ERP in Figure 8(a) moving away from the boundary and cavity.

### Arrhythmogenesis from Focal Stimulus

We have identified that the stark gradients in ERP witnessed around fine-scale anatomical features within the myocardium provide the necessary substrate for arrhythmogenesis upon delivery of a focal stimulus. A correctly-timed stimulus within a high gradient of ERP is expected to cause unidirectional conduction block; tissue will capture and initiate propagation into the direction of low ERP, whilst propagation will not be possible into the direction of higher ERP as the tissue has not fully recovered and cannot be excited. However, unidirectional block does not necessarily directly cause reentry; it is the fact that these regions of high ERP gradient are located in close proximity to structural heterogeneity (vessel cavities, for example), that provide this additional key mechanism. As shown in Figure 9, because unidirectional block occurs close to these physical boundaries, the initial propagation of the captured wavefront is channelled and restricted relatively more than if it had occurred within homogeneous myocardium, using the vessel cavity as a pivot point about which it begins to rotate and reenter. Similar mechanisms, whereby the inherent anatomical complexity restricts the initial movement of the wavefront following unidirectional block would be expected to also be associated with other fine-scale anatomical features, such as trabeculations. Although this example within the idealised model is short-lived, it is expected that when combined with the increased complexity of a bi-ventricular model, such initial reentrant behaviour will be more likely to become sustained, particularly if multiple triggers occur at similar times close by, leading to complex wavefront interactions. Finally, the high magnitude of the ERP gradient within these regions is of critical importance as it provides a relatively large temporal window of vulnerability in which a stimulus may capture and cause unidirectional block which has relevance for the clinical occurrence of focal ectopics.

### Application to Structural Remodelling

Following myocardial infarction, significant structural remodelling may occur leaving highly heterogeneous regions of tissue. As well as consisting of regions of dense fibrosis, infarct scars often contain surviving myocyte bundles which form channels of healthy, excitable tissue through the scar, along with border-zone regions representing the area between viable healthy tissue and non-viable scar that contain an admixture of surviving myocytes and fibrotic scar tissue [3]. The significantly increased heterogeneity within such regions includes the existence of numerous additional intracellular boundaries with fibrotic tissue within the border-zone as well as around the surviving tissue isthmus bundles penetrating the dense scar regions. Previous computational studies have demonstrated how very fine-scale discontinuities representing patchy fibrosis in such regions give rise to significant heterogeneity in tissue activation times and APDs, particularly at fast pacing rates. The resulting induction of discordant APD alternans was seen to lead to heterogeneous conduction block, demonstrating a potentially important cause of reentry initiation due purely to structural heterogeneity during rapid pacing. Here, we uncovered a mechanism of reentry induction following focal stimulus in the presence of structural heterogeneity of primarily a larger-scale form i.e. blood vessels, trabeculations. Thus, the findings uncovered in this study regarding the interaction between electrotonic loading, refractoriness and successful capture of focal ectopics, and the dependence upon immediate structural heterogeneity, may have even more significance under such pathological conditions in the presence of fibrosis.

Recent studies [4], [7] have also demonstrated that the propensity for focal stimuli capture can be enhanced through increases in tissue conductivity anisotropy and by including anisotropic distributions of coupled fibroblasts, both of which affect local electrotonic loading in an anisotropic manner. These conditions are known to occur in the presence of fibrosis such as during infarction and may also therefore play an important role in such circumstances.

### Relation to Physiological Causes of Focal Ectopic Stimuli

The initial focal triggered stimulus, represented here by a ‘forced’ stimulation, may result from a number of physiological processes such as early- or delayed-afterdepolarisations or abnormal automaticity [4], [5]. In this work, we focus on how such a stimulus (however it may be initiated) could bring about successful capture and arrhythmogenesis. Whether these triggers occur more readily under conditions of electrophysiological remodelling, or whether their frequency is in fact unchanged, but changes in electrotonic loading due to structural remodelling (presence of fibrosis and scar) more readily facilitate their successful capture, is currently not understood. We believe the findings from our study provide an important first step, to be later combined with more realistic simulation of the initial trigger along with examining their implications under pathological conditions such as infarction and heart failure. Important to note here is that the key mechanism of arrhythmogenesis uncovered in this study was due to focal stimulus within a region of high ERP heterogeneity, mediated by electrotonic loading changes due to the proximity of fine-scale structural features and tissue boundaries. However, such a mechanism relies on a focal stimulus occurring at the limit of the local ERP (i.e. approximately 160 ms following prior activation). As such, an early-afterdepolarisation would be the more likely cause of such arrhythmogenesis, as a delayed-afterdepolarisation would occur later, at a time outside of the window of local ERP difference (i.e.>200 ms in this case).

### Study Limitations & Future Work

Although a range of electrophysiological metrics were analysed during this work, localised spatial variations in conduction velocity and its restitution were not investigated. ERP is associated with excitability at high pacing rates, and therefore plays an important role in both arrhythmogenesis and the stability of reentrant waves. Thus, restitution effects of conduction velocity and particularly localised differences with respect to structural heterogeneities may prove to provide an important mechanism of arrhythmogenesis and stability due to non-uniform conduction during rapid pacing. In this current study, we have focussed our attention more on arrhythmia initiation due to focal stimulus, and thus we leave the effects of large-scale heterogeneities upon localised conduction velocity in arrhythmias as an interesting avenue of future investigation.

The results presented in this study use a recent rabbit ventricular cell model. However, the extent of electrotonic APD modulation is known to depend upon the membrane resistivity during repolarisation, with different degrees of APD modulation being noted for different species with inherent different membrane electrophysiological properties [29], [30]. Pacing site and overall tissue dimensions are also known to influence the spatial dispersion of APD. The rabbit cell model used here only demonstrates a modest influence of electrotonic loading upon repolarisation and APD. Although we have shown that the dispersion of repolarisation that was present did not significantly influence the spatial distribution of ERP for focal stimulus, caution should be used when extrapolating the findings from this study to other species, for example human, and in the context of pathology, where APD/repolarisation dispersion may be more significant.

## Conclusions

The presence of physiological structural heterogeneity, in the form of intramural vessel cavities and complex endocardial trabeculations, significantly reduces local electrotonic loading during wavefront propagation and focal stimuli. This reduced loading directly affects tissue excitability, increasing upstroke velocity and significantly reducing ERP around fine-scale structures, despite no corresponding significant spatial variation in APD during normal wavefront propagation in the very same region. ERP gradients that exist in the vicinity of such structures provide a mechanism of arrhythmogenesis upon focal stimuli by establishing unidirectional conduction block and combining with the structures themselves to initiate reentry. These fundamental findings may have increased significance under conditions of fibrosis in the presence of extensive tissue and electrophysiological remodelling.
